# Establishment risk of the commercially imported bumblebee *Bombus terrestris dalmatinus*—can they survive UK winters?

**DOI:** 10.1007/s13592-015-0376-8

**Published:** 2015-07-21

**Authors:** Emily L. Owen, Jeffrey S. Bale, Scott A. L. Hayward

**Affiliations:** School of Biosciences, University of Birmingham, Edgbaston, Birmingham, B15 2TT UK

**Keywords:** bumblebee, cold tolerance, rapid cold hardening, native, winter

## Abstract

Bumblebees are regularly exported to countries outside their native range for the purposes of commercial pollination. In contrast to the tight regulations imposed on biological control introductions, the movement of bumblebees has largely been without risk assessment. This study represents the first formal assessment of establishment risk for *Bombus terrestris dalmatinus* in the UK. The ability of workers to survive winter conditions is seen as the primary barrier to establishment, given the year-round colony activity of this sub-species. We use standardised cold tolerance indices as outlined by the EU policy support action ‘REBECA’ as well as assessing rapid cold hardening (RCH) ability. Cold tolerance was comparable to that of the UK-native *Bombus terrestris audax*, including a strong RCH response. Results suggest that *B. t. dalmatinus* could survive mild UK winters in southern areas and potentially displace *B. t. audax*. The implications of ongoing climate change on establishment risks are discussed.

## Introduction

Given the economic importance of insect pollination (Gallai et al. 2009) and the decline of the north American honeybee, *Apis mellifera*, (Tentcheva et al. 2004), alternative pollinators have increasingly been used to enhance the yield and quality of commercially valuable crops. In the past 50 years, demand for commercial pollination has increased by 300 %, yet there has only been a 45 % increase in managed honey bee hives (Aizen et al. 2008). Due to improvements in mass rearing methods, bumblebees have been employed as commercial pollinators since 1988 (Inari et al. 2005) and are now the sole pollinators of certain crops such as tomatoes. In 2004, 40,000 ha of greenhouse tomatoes (*Lycopersicon esculentum*) worldwide were pollinated by bumblebees, with an estimated value of €12,000 million (Velthuis and van Doorn 2006). Bumblebees are also deployed in the pollination of alfalfa (*Medicago sativa*), clover (*Trifolium* spp.), oilseed rape (*Brassica napus*), brown mustard (*Brassica juncea*), sunflower (*Helianthus annuus*) and fruits such as strawberry (*Fragaria* × *ananassa*), melon (*Cucumis melo*) and kiwifruit (*Actinidia deliciosa*) (Goulson 2010). Key to their success is the ability to ‘buzz pollinate’ (Buchmann 1983) by moving their flight muscles rapidly, causing the flower and anthers to vibrate which dislodges pollen and enhances the efficiency of pollen harvesting 400-fold (Winter et al. 2006).

The most widely used bumblebee in a commercial setting is *Bombus terrestris dalmatinus* (Ings et al. 2005a), due to the large size of workers and high success rate of colonies, e.g. easy commercial production of large colony numbers, and benefits for farmers including lower production costs, increased yields and improved fruit quality (Velthuis and van Doorn 2006). This subspecies is native to South East France, Northern Italy, the Balkanic Peninsulas, Turkey and North Iran, (Rasmont et al. 2008). However, approximately one million colonies were transported across 57 countries in 2006, 16 of which are outside its native range (Ings 2006). Because precautions were not undertaken to assess establishment risk (Velthuis and van Doorn 2006), establishment occurred in Japan, Chile, Argentina, Israel, New Zealand and Tasmania (McFadyen and Lloyd 2006; Goka 2010). There was also concern regarding potential competitive displacement of native bee species, heightened in areas of sparse floral resources (Ings et al. 2005b; Goulson 2003). Studies of exotic invaders competing with native species abound within the literature (Radville et al. 2014) and can occur rapidly where there is considerable overlap in habitat and other resource requirements (Short and Petren 2012)—which is often true of competing *Bombus* spp. (Dohzono and Yokoyama 2010). There are many well-documented examples for bees, including introduced *A. mellifera* outcompeting native solitary and honey bees on Tenerife (Dupont et al. 2004). While in Tasmania, foraging by native solitary bees is prevented by the presence of non-native *B. terrestris* (Hingston and McQuillan 1999). Competition for nest sites between native bees and *B. terrestris* can further decrease species richness and abundance (Hingston and McQuillan 1999; Goulson 2003).

Commercial colonies of *B. t. dalmatinus* are regularly imported into the UK, despite having a native population of *Bombus terrestris audax*. Control measures to prevent establishment have only been recently introduced, with ‘queen excluders’ (devices which prevent queens from exiting colonies) mandatory since January 2013 (Natural England 2013). However, given the scale of importation to the UK (10,000 colonies annually), it is possible that some *B. t. dalmatinus* queens may have escaped. To date, no formal assessment of establishment risk has been undertaken in the UK, although there is emerging evidence that *B. t. dalmatinus* may have become established in the southern UK (Ings et al. 2006). This is of concern because Ings et al. (2006) found consistently higher nectar foraging rates and an increased production of gynes in *B. t. dalmatinus*, suggesting that it could outcompete the native *B. t. audax*.

The life cycle of *B. terrestris* in Northern Europe has been extensively researched and is well understood (Goulson 2010). In early spring, mated overwintered queens emerge from diapause (Rasmont et al. 2008), establish a nest and feed their first brood of larvae with pollen to initiate colony development (Beekman and van Stratum 2000). Emerging workers then tend to all further eggs (Sladen 1912). Egg laying continues until late summer when new diploid queens and haploid males are produced (Gadau et al. 2001). New queens then accumulate glycogen reserves, mate, locate suitable hibernacula and enter the ‘diapause’ state (Alford 1969). Males and remaining workers within the colony die at the onset of winter. This is in contrast to *B. t. dalmatinus* which, in their native Mediterranean environment, typically remain active throughout the year, except for a period of summer aestivation—although a winter diapause has been documented in alpine regions of Turkey (Gurel et al. 2008). An important barrier to the establishment of non-native species in regions with a seasonal climate is winter cold (Bale and Hayward 2010). Thus, the phenology of *B. t. dalmatinus* may be maladapted to the UK climate by producing winter-active colonies instead of queens entering diapause. Interestingly, climate warming appears to have driven *B. t. audax* towards producing winter-active colonies in the southern UK (Stelzer et al. 2010), and active workers could potentially survive mild UK winter conditions (Owen et al. 2013). This has never been assessed for *B. t dalmatinus* but is key to determining whether this sub-species could become established and possibly displace the native *B. t. audax*? The life expectancy of winter colonies would then depend on temperatures not dropping below the tolerance limits of workers and queens as well as access to forage. With good evidence that several important pollen sources persist throughout UK winters in urban environments (Ings et al. 2006), understanding the physiological limits of winter survival is extremely important.

There exists an extensive literature on insect cold tolerance and its application in assessing the establishment risk of introduced species (Hatherly et al. 2005; Hughes and Bale 2009; Hughes et al. 2010; Coombs and Bale 2014). Indeed, these assessments form the basis of commercial licensing to release biocontrol agents in several EU countries (REBECA 2014). Ecologically relevant indices of cold tolerance are used to inform the likelihood of establishment of an insect, including the use of lethal time (LTime) and lethal temperature (LTemp) experiments(Owen et al. 2013), which assess survival after exposure to acute and chronic low temperatures. In the current study, we use the same indices to determine the likelihood of establishment of the non-native bumblebee *B. t. dalmatinus* in the UK. In addition, we assess the capacity of winter-active workers to respond rapidly to environmental variability through a process of rapid cold hardening (RCH), which is also important for winter survival (Lee et al. 1987). We directly compare the cold tolerance of *B. t. dalmatinus* with *B. t. audax* and determine the likelihood of establishment of *B. t. dalmatinus* in the UK.

## Materials and methods

### Culture system

Mature colonies of *B. t. dalmatinus* were obtained from Biobest NV (Westerlo, Belgium) and maintained at 20 °C in constant darkness. Bees were manipulated under red illumination to minimise disturbance (Sadd 2011). Nectar was available within the colony using a wick system connected to a reservoir of BioGluc^®^ nectar, and pollen paste was available ad libitum (Biobest NV). In total, 19 colonies were used, delivered in batches of two to five at regular intervals in 2013 (22 April, 15 May, 26 July, 22 August and 22 October). Preliminary experiments were conducted to determine whether individuals from each colony within a batch demonstrated significant differences in cold tolerance phenotypes: none did. Subsequently, individuals were selected randomly across all colonies within a batch for use in each experimental treatment (including controls). For each experimental treatment, *n* = 30 worker bees were used, unless otherwise stated. Control samples of 30 bees were exposed to 15 °C for the maximum experimental duration, and survival (determined as ability to move) was assessed after 72 h.

### Lethal temperature

Bumblebees were placed into six test tubes (*n* = 5 per tube) containing type K exposed wire thermocouples to record body temperature. Tubes were placed into an alcohol bath (Haake Phoenix 11 P2, Thermo Electron Corporation), programmed to cool from 20 °C, at a rate of 0.2 °C min^−1^, to a range of sub-zero temperatures between −5 and −8 °C. Bumblebees were held at each temperature for 15 min before the temperature was increased back to 20 °C at the same rate and survival assessed as described earlier.

### Lethal time

Workers were added in groups of five to six conical flasks (25 mL Pyrex) and placed inside a Fryka^®^ (B30 Cold Box, Fryka, Germany) incubator set at 0 °C for a range of durations, between 2 and 11 days (without access to food). Before and after each exposure, bees were held at 10 °C for 1 h to prevent the possibility of cold and heat shock mortality, respectively. Bumblebees were removed from the incubator, added to a recovery box, and survival was assessed as previously described.

### Rapid cold hardening

Determination of the discriminating treatment was undertaken at −5 °C, as this was the lowest sub-zero temperature which induced mortality, whilst having no incidence of freezing (supercooling points (SCPs) ranged from −5.1 to −10.1 °C). Worker bees were taken from their rearing temperature (20 °C), added to test tubes with thermocouples as previously described and placed directly in an alcohol bath set at −5 °C for a range of durations (2, 4, 6, 8 and 10 h; six replicates of *N* = 5 per treatment). Bees were then re-warmed to rearing temperature at a rate of 0.2 °C min^−1^, and survival was assessed. The shortest time duration that resulted in between 10 and 20 % survival was selected as the discriminating treatment (Lee et al. 1987).

To assess the RCH response, bees were added to test tubes as previously described and exposed to one of two RCH regimes prior to transfer to the discriminating treatment: 1 h at 0 °C or gradual cooling at 0.2 °C min^−1^ to −5 °C (six replicates of *N* = 5 per treatment). Re-warming to 20 °C and survival assessment were as previously described after 72 h. Evidence of RCH was determined by any increase in survival relative to direct transfer to the discriminating treatment.

### Impact of RCH on supercooling point

After a period of 1 h at 0 °C, the SCPs of 30 workers were measured, using established methods (see Hughes and Bale 2009). Briefly, bees were inserted individually into test tubes containing type K exposed wire thermocouples and placed in an alcohol bath programmed to cool from 20 °C to −20 °C at a rate of 0.2 °C min^−1^, and freezing exotherms were detected via a computerised recording system.

### Statistical analysis

All results were tested for normality using a Kolmogorov-Smirnov test. LTemp_10,50,90_ and LTime_10,50,90_ experiments were analysed via Probit analysis (Finney 1971) in Minitab^®^ to identify the temperature at which 10, 50 or 90 % mortality occurred. RCH experiments were non-normally distributed, and so, independent samples Kruskal-Wallis tests with pairwise comparisons were undertaken on SPSS^®^. RCH and SCP results were compared via a one-way ANOVA on Minitab^®^.

## Results

### Lethal temperature

**Figure 1.**
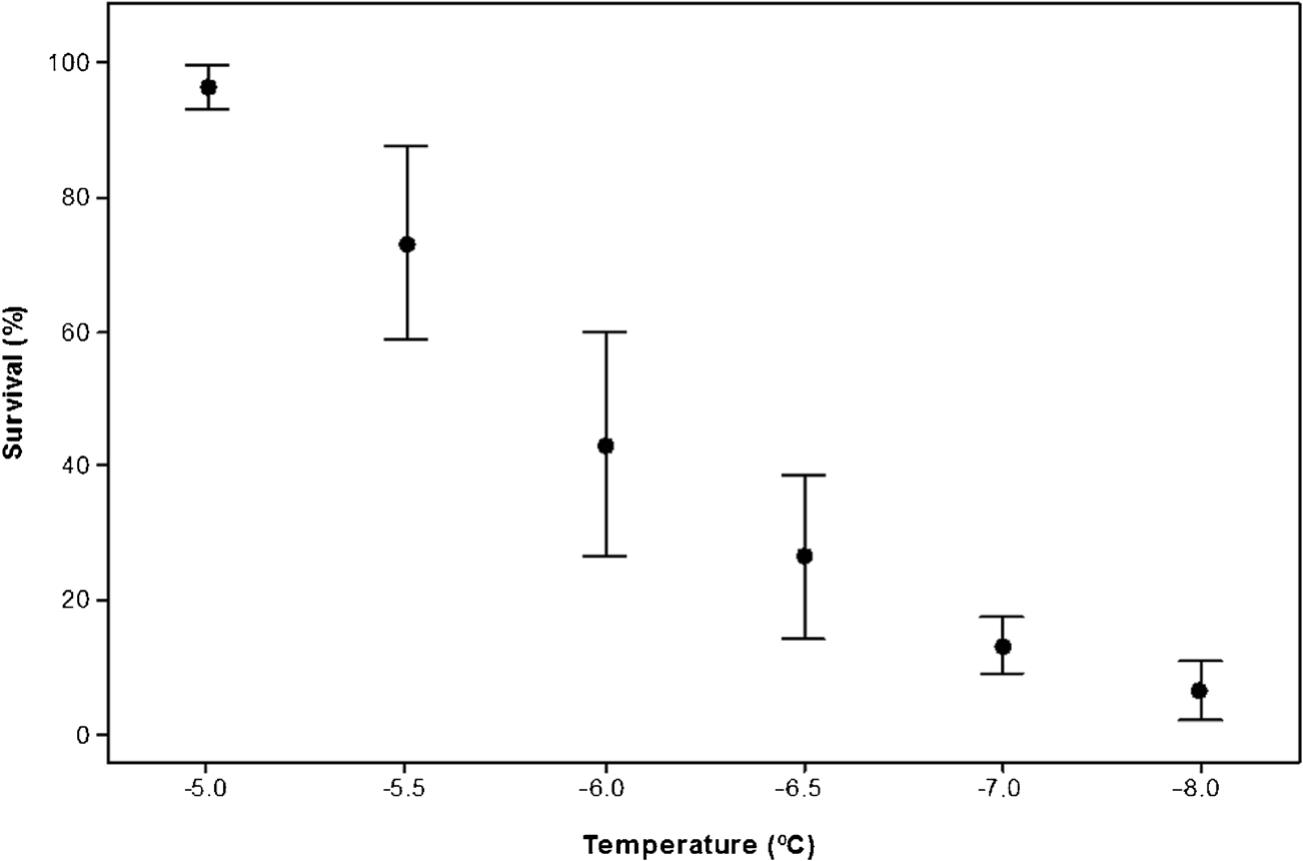
Survival of worker bumblebees (*B. t. dalmatinus*) held at a range of sub-zero temperatures for 15 min. Each point represents six replicates of five bees ± SEM.

Most *B. t. audax* workers (96.6 ± 3.3 %) survived 15 min at −5 °C, but this decreased to just 6.7 ± 4.2 % survival following 15 min at −8 °C (Figure 1). Probit analysis determined that LTemp_10,50,90_ temperatures were −4.6 ± 1.0, −6.1 ± 1.0 and −7.3 ± 3.7 °C, respectively (regression *P* < 0.001, Pearson’s goodness-of-fit *χ*^2^ = 15.4572, *df* = 4 *P* = 0.004). There was no mortality in the control sample.

### Lethal time

**Figure 2.**
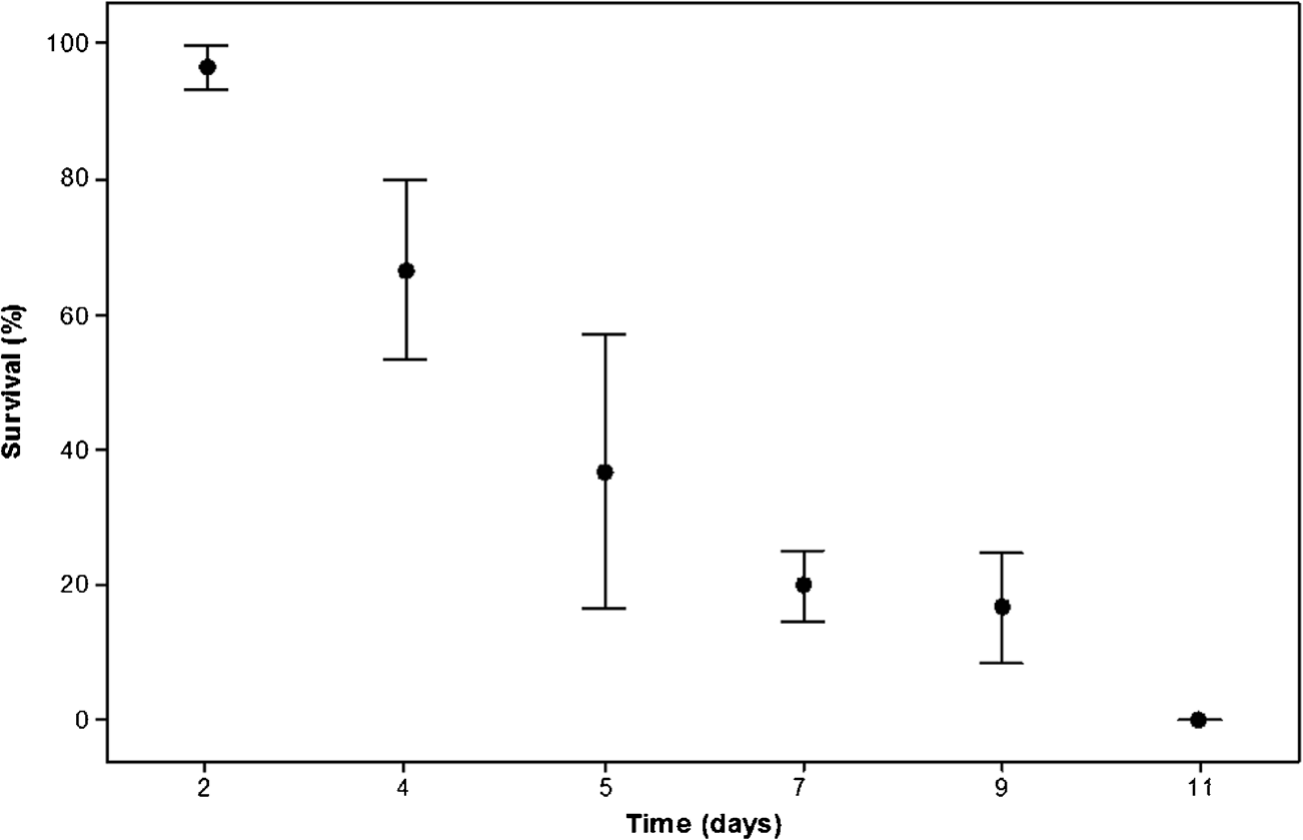
Survival of worker bumblebees (*B. t. dalmatinus*) at a range of durations at 0 °C. Each point represents six replicates of five bees ± SEM.

Worker survival was 96.7 ± 3.3 % following 2 days at 0 °C but declined rapidly to 20 ± 5.2 after 7 days at 0 °C (Figure 2). After 11 days, survival was 0 %. Probit analysis determined LTime_10,50,90_ to be 2.5 ± 1.1, 4.8 ± 1.1 and 9.2 ± 1.1 days, respectively (regression *P* < 0.001, Pearson’s goodness-of-fit *χ*^2^ = 2.6, *df* = 4 *P* = 0.62). There was no mortality in the control sample.

### Rapid cold hardening

**Figure 3.**
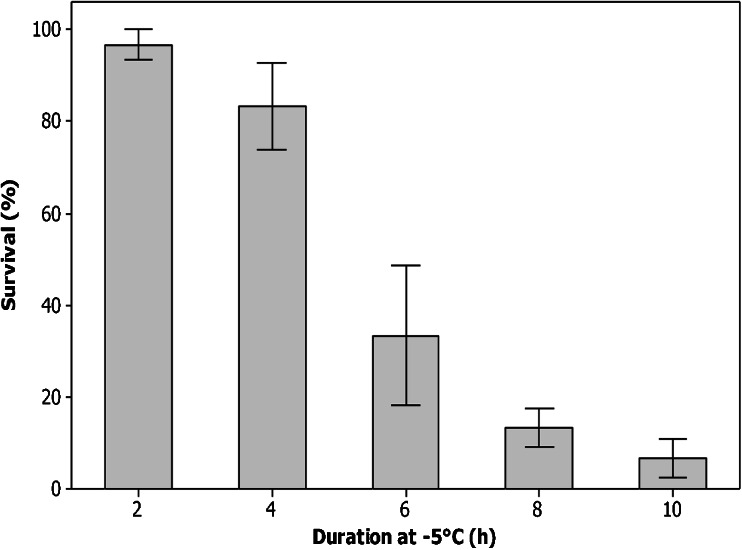
Determination of a discriminating treatment for rapid cold hardening in worker bumblebees (*Bombus terrestris dalmatinus*). Mean survival (±SEM) of worker bumblebees exposed to periods of 2, 4, 6, 8 and 10 h at −5 °C, *n* = 30 per temperature.

Mean survival of workers at −5 °C decreased with increasing duration of cold exposure, from 96.7 ± 3.3 % after 2-h exposure, to 6.7 ± 4.2 % after 10-h exposure (Figure 3); 8 h at −5 °C was chosen as the discriminating treatment (13.3 ± 4.2 %). An independent samples Kruskal-Wallis test (*H* = 20.7, *df* = 4) with pairwise comparisons indicated that survival at 2 and 10 h, 4 and 10 h and 2 and 8 h was significantly different (*P* < 0.01, <0.05, <0.05, respectively).

Both pre-treatments induced a strong RCH response with 100 % survival in workers cooled at 0.2 min^−1^ or exposed to 1 h at 0 °C prior to being placed at −5 °C for 8 h. This was significantly higher than survival as a result of direct transfer to −5 °C for 8 h (*H* = 22.6, *df* = 3, *P* < 0.01).

A period of 1 h at 0 °C was sufficient to significantly decrease (*P* < 0.01) the SCP of workers, compared to controls (Table I). This suggests that RCH has the capacity to lower the SCP of workers and provides a mechanism for reducing freezing risk after a RCH pre-treatment at 0 °C.

**Table I:** Impact of rapid cold hardening on supercooling point.

Treatment group	N	Mean ± SE (°C)	Range (°C)
SCP control	30	−7.1 ± 0.2*	−5.1 to −10.1
SCP following RCH	30	−8.4 ± 0.2*	−5.6 to −12.2

The supercooling points of workers exposed to a pre-treatment of 1 h at 0 °C versus controls

*Significant (*F* = 12.4, *df* = 59 *P* < 0.01)

## Discussion

Characterising the ability of non-native terrestrial invertebrates to survive winter is deemed critical to assessing establishment risk at temperate latitudes (Bale and Hayward 2010). There is a considerable body of work addressing this issue for glasshouse biocontrol agents (Hatherly et al. 2005; Hughes and Bale 2009; Hughes et al. 2010; Coombs and Bale 2014), but far less attention has been paid to commercial pollinators. Commercial colonies of *B. t. dalmatinus* were first imported to the UK in 1989 (Kwon 2008), with no restrictions on their use until 2013 (Ings et al. 2005a). This led to concerns regarding establishment risk (Ings et al. 2006), and since 2013, *B. t. dalmatinus* has only been licensed for pollination in glasshouses and polytunnels in the UK, as well as colony boxes being fitted with queen excluders (Natural England 2013). However, clearly, some queens may have escaped prior to these restrictions coming into place. Given that this subspecies is typically active year round, it is pertinent to assess the ability of workers to survive UK winter conditions. This will formally assess establishment risk using the same criteria as other non-native insects and provide insight regarding whether *B. t. dalmatinus* might displace native *B. t. audax*.

**Figure 4.**
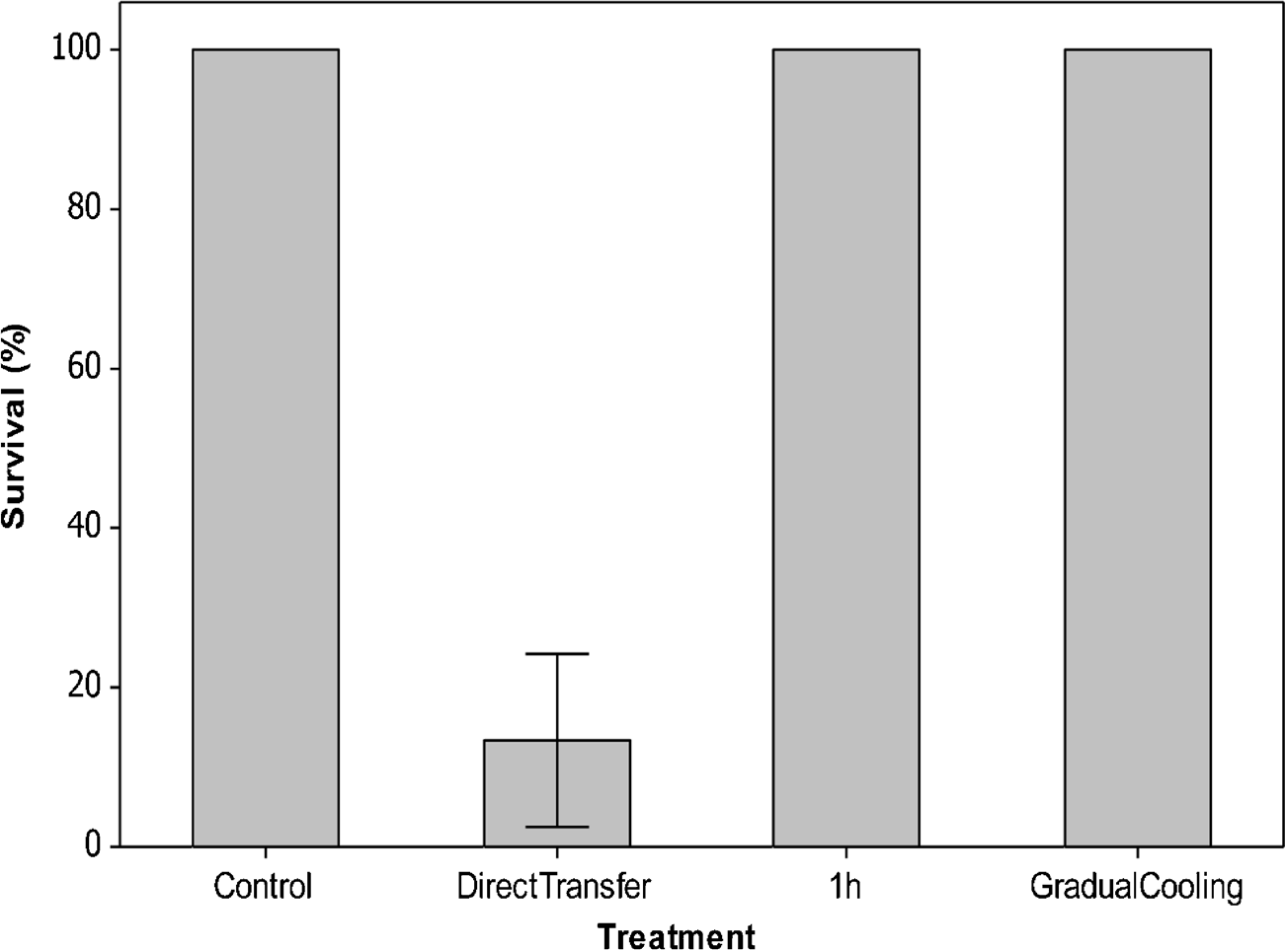
A rapid cold hardening response in worker bumblebees (*Bombus terrestris dalmatinus*). Mean survival (±SEM) of worker bumblebees after direct transfer to the discriminating treatment (−5 °C for 8 h), a pre-treatment of 1 h at 0 °C or gradual cooling (at 0.2 min^−1^) to the discriminating treatment. Each point represents six replicates of five bees ± SEM.

This study has identified that *B. t. dalmatinus* is freeze avoiding (see Bale 1996), with limited supercooling ability (mean SCP of workers −7.1 ± 0.2). This is comparable to the solitary bee *Megachile rotundata* (−8 °C; Sheffield 2008) as well as *B. t. audax −*7.1 ± 0.2 °C (Owen et al. 2013). Despite this limited supercooling ability, workers were able to tolerate exposures to temperatures close to their SCP for short periods, e.g. 97 ± 3.3 % survived exposure to −5 °C for 2 h (Figure 3). Survival declined dramatically at temperatures below −5 °C, however, with 50 % mortality at −6.1 ± 1.0 °C, and it is likely that this was the result of freezing rather than cold shock. This level of cold tolerance is very similar to *B. t. audax* workers (Owen et al. 2013), suggesting a comparable ability to survive winter conditions outside the colony for short periods. To survive the entire winter, however, workers must forage to provision the colony. While there is good evidence that urban areas in parts of the UK provide sufficient floral resources over winter (Ings et al. 2006), foraging will expose workers to potentially rapid temperature fluctuations, for example, 0.03 °C min^−1^ in Birmingham during winter 2011 (unpublished data). We identified a very strong RCH response in *B. t. dalmatinus* (Figure 4), which is again comparable to *B. t. audax* (Owen et al. 2013), and this could be important for winter survival. For example, if foraging workers were unable to return to the colony at night, they would be exposed more extreme sub-zero temperatures, e.g. −7.7 °C minimum temperature in Birmingham during February 2014 (Met Office 2014). Low temperatures of this nature have the potential to cause significant freezing mortality (LTemp_90_ = −7.3 °C); however, because RCH significantly lowered the SCP (Table I) and enhanced the cold tolerance of *B. t. dalmatinus* (Figure 4), the risk of freezing and/or pre-freeze mortality is reduced. Processes underpinning SCP reduction in other insects are thought to include the accumulation sodium chloride, urea, glycerol or glucose (Wilson et al. 2003), but this is yet to be studied in any bee species as *B. t. dalmatinus* and *B. t. audax* (Owen et al. 2013) are the only two Hymenoptera species for which RCH has been recorded to date. Lowering of the SCP during RCH is in contrast with several other insect species, for example, the Antarctic midge, *Eretmoptera murphyi* (Everatt et al. 2012), and house fly, *Musca domestica* (Coulson and Bale 1990). The capacity to RCH is not uncommon in Mediterranean species, however, and is thought to contribute to establishment potential (Nyamukondiwa et al. 2010).

Winter-active bees will, of course, not only be exposed to brief periods of sub-zero temperatures during foraging trips. They must also survive chronic cold within the colony. Subterranean temperatures often remain around 0 °C throughout winter (UK Environmental Change Network 2013), and while bumblebees possess the ability to thermoregulate their colonies to between 30 and 32 °C (Heinrich 1975), a lack of winter floral resources or an excessive thermoregulatory demand may mean that colonies are unable to consistently maintain a favourable temperature (Moret and Schmidt-Hempel 2000). This means that colony temperatures may equilibrate to the temperature of the surrounding earth for extended periods. *B. t. dalmatinus* workers experienced very little mortality following 2 days at 0 °C (Figure 2), but this increased to 50 % after just 4.8 ± 1.1 days. These results are in line with LTime values for *B. t. audax*, with 50 % mortality after 7.2 ± 1.1 days (Owen et al. 2013). Whether this is a direct result of prolonged chilling or also a consequence of starvation needs to be further investigated, but metabolic rates were likely very low at 0 °C unless workers were actively trying to increase their body temperature during the treatment. Either way, winter-active colonies of both subspecies are likely to suffer significant mortality during typical northern European winters unless they are able to invest resources in maintaining colony temperature. However, this represents a significant thermoregulatory demand and would be predicted to negatively impact colony longevity, growth and subsequent production of new queens (Weidenmüller et al. 2002). Recent UK winters certainly appear to have been too cold for *B. t. audax* colonies to sustain colony development to the point of producing new queens, although worker numbers did persist for over 2 months of winter (2012/13) in Birmingham, UK (Owen et al., unpublished data). Given the comparable cold tolerance of *B. t. dalmatinus*, a similar fate for winter-active colonies of this sub-species seems likely, indicating that a winter diapause strategy has a selective advantage under current conditions.

Climate change may further facilitate the establishment of *B. t. dalmatinus*, with estimated increases in global surface temperature of between 1 and 3.5 °C by 2100 (Cannon 2004). Thus, in areas where both sub-species are winter active, the comparable cold tolerance of workers, combined with the larger size and superior foraging performance of *B. t. dalmatinus* (Ings et al. 2006), suggests that the non-native may outcompete *B. t. audax.* Competition is known to be heightened in areas of patchy floral resources (Goulson 2003), and phenological mismatching between plants and pollinators is predicted to exacerbate this problem (Schweiger et al. 2010). Under milder conditions, therefore, winter-active *B. t. dalmatinus* may be more effective at producing queens for the following spring. That said, a lack of diapause, even under favourable conditions, can have a negative impact on subsequent colony characteristics (Beekman and Van Stratum 2000), and this would require further investigation. There is also evidence that climate change might bring more frequent extreme cold events during winter (Rosenzweig et al. 2001), and under this scenario, it may be *B. t. audax* that has the advantage. This is because diapausing queens will be better able to survive than winter-active colonies. As outlined above, our own studies in the West Midlands indicate that neither sub-species could have survived recent winters as active colonies (Owen et al., unpublished data), so it is possible that a north-south divide may emerge defined by the ‘decision’ to enter diapause or not—with *B. t. dalmatinus* more successful in the south and *B. t. audax* at an advantage in the north. A similar driver of species distribution limits has been noted in the green stink bug, *Nezara viridula*, in Japan, where the timing of diapause induction determines the northern limit of winter survival (Musolin and Numata 2003). Given the unpredictability of year on year climate patterns, however, the key message from this study is the importance of conducting formal establishment risk assessments before releasing non-native insects, even if the subject is considered a beneficial pollinator.
